# Lerodalcibep: Another Crucial Addition to the Dyslipidaemia Arsenal

**DOI:** 10.31083/RCM42070

**Published:** 2025-09-30

**Authors:** Amirhossein Sahebkar, Sercan Karav, Wael Almahmeed, Tannaz Jamialahmadi

**Affiliations:** ^1^Center for Global Health Research, Saveetha Medical College and Hospitals, Saveetha Institute of Medical and Technical Sciences, Saveetha University, 602105 Chennai, India; ^2^Biotechnology Research Center, Pharmaceutical Technology Institute, Mashhad University of Medical Sciences, 9177948954 Mashhad, Iran; ^3^Applied Biomedical Research Center, Basic Sciences Research Institute, Mashhad University of Medical Sciences, 9177948564 Mashhad, Iran; ^4^Department of Molecular Biology and Genetics, Canakkale Onsekiz Mart University, 17100 Canakkale, Turkey; ^5^Heart and Vascular Institute, Cleveland Clinic Abu Dhabi, 112412 Abu Dhabi, United Arab Emirates; ^6^Pharmaceutical Research Center, Pharmaceutical Technology Institute, Mashhad University of Medical Sciences, 9177948954 Mashhad, Iran

The recent comprehensive review in the *Reviews in Cardiovascular Medicine* highlights various therapeutic agents for dyslipidaemia [1], but notably, the inclusion of lerodalcibep is crucial, given its promising efficacy and safety profile. Lerodalcibep, a promising proprotein convertase subtilisin kexin-9 (PCSK9) adnectin, inhibits the binding of PCSK9 to low-density lipoprotein (LDL) receptors, preventing their degradation and consequently lowering LDL cholesterol (LDL-C) levels [2]. This mechanism is particularly beneficial for patients at high risk of atherosclerotic cardiovascular disease (ASCVD). In the 52-week LIBerate-HR trial, lerodalcibep demonstrated substantial efficacy in reducing LDL-C (60.3%) in patients with or at high-to-very high risk of ASCVD [2]. In the LIBerate-HeFH trial involving participants with heterozygous familial hypercholesterolemia, treatment with lerodalcibep for 24 weeks led to a substantial 58.6% reduction of LDL-C levels, with 68% of participants achieving an LDL-C reduction of at least 50% while meeting the European Society of Cardiology targets [3]. Importantly, the long-term LIBerate-OLE trial showed that monthly lerodalcibep administration reduced LDL-C by >60%, without any evidence of attenuation even after >2 years [4]. Lerodalcibep, administered subcutaneously, presents a comparable safety profile to placebo, which is essential for patient compliance and long-term care. Compared to monoclonal antibodies, the structure of lerodalcibep, which combines adnectin and human serum albumin, allows for smaller injection volumes, less frequent injections, and improved stability at room temperature. The legacy effect highlighted in the review underscores the need for sustained lipid-lowering therapy in preventing cardiovascular events. Consequently, integrating lerodalcibep into therapeutic options represent a significant enhancement to the treatment repertoire for attaining optimal cholesterol levels in patients who are statin-intolerant or need further LDL-C reduction.
